# Analysis of Flavonoids in *Rhamnus davurica* and Its Antiproliferative Activities

**DOI:** 10.3390/molecules21101275

**Published:** 2016-09-23

**Authors:** Guilin Chen, Xun Li, Flora Saleri, Mingquan Guo

**Affiliations:** 1 Key Laboratory of Plant Germplasm Enhancement and Specialty Agriculture, Wuhan Botanical Garden, Chinese Academy of Sciences, Wuhan 430074, China; cjl1652009@163.com (G.C.); lixunyiyi@126.com (X.L.) didiiflora@gmail.com (F.S.); 2 Graduate University of Chinese Academy of Sciences, Beijing 100049, China; 3 Sino-Africa Joint Research Center, Chinese Academy of Sciences, Wuhan 430074, China

**Keywords:** *Rhamnus davurica*, fingerprint profile, flavonoids, antiproliferative activities, HPLC-ESI-MS/MS

## Abstract

*Rhamnus davurica* Pall. (*R. davurica*) has been used as a traditional medicinal herb for many years in China and abroad. It has been well documented as a rich source of flavonoids with diversified structures, which in turn results in far-ranging biological activities, such as anti-inflammation, anticancer, antibacterial and antioxidant activities. In order to further correlate their anticancer potentials with the phytochemical components, the fingerprint profile of *R. davurica* herb from Dongbei was firstly investigated using HPLC-ESI-MS/MS. Thirty two peaks were detected and identified, 14 of which were found in *R. davurica* for the first time in this work. Furthermore, a total of 23 peaks were resolved as flavonoids, which are the major components found in *R. davurica*. Meanwhile, the antiproliferative activities against human cancer cells of HT-29 and SGC-7901 in vitro exhibited distinct inhibitory effects with IC_50_ values at 24.96 ± 0.74 and 89.53 ± 4.11 μg/mL, respectively. Finally, the general toxicity against L-O2 cells displayed a much higher IC_50_ at 229.19 ± 8.52 μg/mL, which suggested very low or no toxicity on hepatic cell viability. The current study revealed for the first time the correlations between the flavonoids of *R. davurica* with their antiproliferative activities, which indicated that the fingerprint profile of flavonoids and their anticancer activities could provide valuable information on the quality control for herbal medicines and their derived natural remedies from this valuable medicinal plant.

## 1. Introduction

Flavonoids are one of the most ubiquitous polyphenolic secondary metabolites existing in natural products, which are widely found in plants and their corresponding derived foods. In plants, the basic chemical skeleton of flavonoids is biosynthesized by a series of condensation reactions between three malonyl residues (A ring) and hydroxycinnamic acid (B ring), namely a C6-C3-C6 skeleton [1]. These secondary metabolites could be classified into several types of structures according to the variations in the aglycones, sugar moieties and intersaccharide linkage [2]. Flavonoids play important roles in plant ecology and physiology, and many plants being rich in flavonoids have been used as herbal medicines or functional foods for thousands of years [3]. Recent studies showed that flavonoids and their glycoconjugates have exhibited remarkable pharmacological and biological properties, including anti-inflammatory, antioxidant, antitumor, antiallergic, antibacterial, antiarteriosclerotic and antiestrogenic activities [4,5,6]. To date, more than 8000 natural flavonoids have been identified with diversified activities, and it has been reported that their discrepant biological activities are mainly due to their structural diversity and various chemical modifications, such as methylation, hydroxylation, acylation and glycosylation [7].

The genus *Rhamnus* (Rhamnaceae) is known to be rich with phenolic substances, such as flavonoids, anthraquinones and naphthalenes [8,9,10]. Structural analysis also revealed that the aglycones of the flavonoids are mainly quercetin, kaempferol, isorhamnetin, aromadendrin, taxifolin, rhamnocitrin, rhamnazin, and so on [11,12,13]. Furthermore, several types of constituents, including flavonoids, anthraquinones, naphthols, anthrone, triterpenes and their glycosides, have been isolated from *Rhamnus* species [14,15].

*Rhamnus davurica* Pall. (Rhamnaceae), a shrub or small tree, is mainly distributed in the Northeast of China (Dongbei) and commonly grows in the valleys and the mountain slopes. The medicinal parts of *R. davurica* are the barks, leaf and seeds, which have long been consumed traditionally as a kind of folk remedy for the treatment of dysuresia, pruritus and constipation in China and other Asian countries [16]. In recent years, a number of pharmaceutical research works have proven the potential anti-inflammatory [17], immunomodulation [18] and anti-allergic activities [19] of crude extracts from *R. davurica*. Besides, the ethyl acetate (EA) extract of the fruits of *Rhamnus nepalensis* Laws., obtained in Vietnam, showed significant cytotoxicity to the human oral carcinoma (KB) cell line [14]. Nevertheless, the phytochemical investigation and its associated potential antineoplastic effects of *R. davurica* still remain unclear up to now. Generally, plant-based herbal medicines in China have long been used for treating a number of ailments in the form of crude extracts. Pharmacological experiments further revealed that antioxidants exhibited strong abilities to reduce the oxidative damage in tissues, which is a pivotal etiological factor involved in many chronic human diseases, such as cancers, and thus, flavonoids have been considered as promising antineoplastic substances due to their remarkable antioxidant and free radical scavenging properties [6,10,20,21]. As a consequence, a great deal of attention has been paid to identifying and characterizing those flavonoids from natural resources. In this regard, it is important to thoroughly figure out the chemical components from *R. davurica* and their associated antiproliferative activities.

Fingerprint analysis of bioactive compounds in crude extracts from medicinal plants has attracted widespread interests and promoted a much better understanding of traditional herb medicines in recent years. Currently, high performance liquid chromatography coupled with electrospray ionization tandem mass spectrometry (HPLC-ESI-MS/MS) has been widely used for the fast separation and structural identification of flavonoids and their glycosides without tedious isolation and purification of pure compounds from the complex natural products [22]. Not only could this powerful technique provide rich information on the molecular mass, molecular formulas and fragment ions, but also can offer distinguishing the types of *O*-glycosides, *C*-glycosides and *O*, *C*-glycosides, as well as the position of the substituents [2,3,23]. In the present study, the fingerprint of flavonoid constituents in *R. davurica* was firstly determined using HPLC-ESI-MS/MS, and then, the quantitative analysis of these components was conducted by comparisons of their peak areas with the corresponding standards. Furthermore, as a part of our ongoing research for the discovery and development of natural anti-tumor constituents from *R. davurica*, the antiproliferative activities against human cancer cells including colon carcinoma (HT-29) and gastric carcinoma (SGC-7901) in vitro were conducted to evaluate the antineoplastic effects. The general toxicity on normal human hepatic cell (L-O2) had also been carried out to estimate the cytotoxicity. To the best of our knowledge, the current study revealed for the first time the correlations between the flavonoids in *R. davurica* with their antiproliferative activities. Meanwhile, the fingerprint profile of flavonoids and their anticancer activities could provide valuable information on the quality control for *R. davurica* and the derived natural remedies from this valuable medicinal plant.

## 2. Results and Discussion

### 2.1. Optimization of Chromatographic Conditions and HPLC Fingerprint Profile of R. davurica (Dongbei)

A number of previous studies were carried out to optimize the HPLC analytical conditions for the separation of diverse flavonoids in the *Rhamnus* extracts [11]. For the systematic investigations, the HPLC method was optimized to reduce run-time while maintaining the chromatographic separation efficiency and peak shape. Besides, acetonitrile (ACN) was selected as the organic phase due to its lower background and higher sensitivity than that of methanol. Furthermore, formic acid (0.1%, *v*/*v*) was applied as an additive in the mobile phase, which could facilitate better resolution by reducing the peak tailing [24,25]. Due to the structural properties of the flavonoid compounds, the characteristic wavelength of 360 nm was chosen for the detection [26]. At last, the final optimized conditions were obtained within 35 min by using a Waters Sunfire RP-C18 column and a binary elution gradient of ACN/water (0.1% FA).

**Figure 1:**
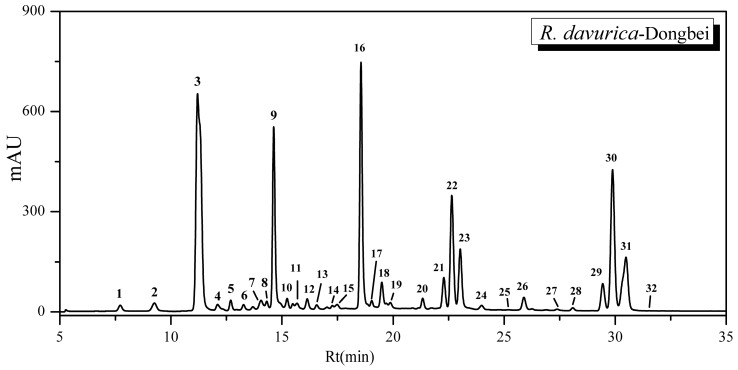
The HPLC-UV chromatogram of *R. davurica* (Dongbei) at 360 nm using the optimized analytical method. The peak numbers in this figure correspond to those used in Table 1.

After extracted three times from the barks of *R. davurica*, the components of flavonoids were further enriched by a polyamide column and then analyzed with the optimized HPLC conditions (Section 3.5.1). The HPLC fingerprint profile of the phytochemical components from *R. davurica* at 360 nm is shown in Figure 1. In more detail, a total of 32 peaks were resolved under the optimal chromatographic conditions, which are subsequently summarized in Table 1 according to their corresponding peak numbers.

### 2.2. Structural Identifications of Flavonoids Using HPLC-ESI-MS/MS Analysis

**Figure 2:**
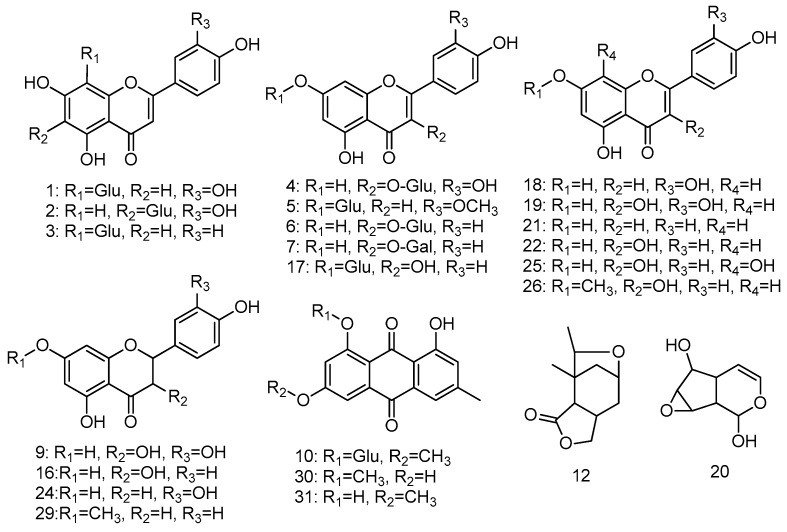
The chemical structures present in *R. davurica*. The peak numbers in this figure correspond to those used in Table 1.

According to the HPLC-UV chromatogram of *R. davurica* shown in Figure 1, 32 components were detected and are summarized in Table 1. Structural identifications and characterizations were successfully carried out based on the comparisons of their ESI-MS/MS data with the corresponding standards and fragmentation pathways reported in the previous literature. Based on the identifications, the corresponding chemical structures present in *R. davurica* are shown in Figure 2. Twenty three compounds out of 32 are flavonoids and their derivatives, which consisted of the major components of *R. davurica*. Considering that the different types of aglycones, the sites of glycosylation and other substituents and the corresponding fragmentation pathways all contribute to the structural diversities, those detected components were further divided into four groups in order to simplify the MS/MS illustrations, including flavone aglycones, flavonoid glycosides, anthraquinones and others. The ESI-MS/MS analysis of those components was conducted in negative ion mode, and their retention times (Rt), calculated molecular masses and MS/MS data are shown in Table 1, respectively. The identifications, interpretations and verifications of these compounds are discussed thereafter in more detail.

#### 2.2.1. Identification of Flavone Aglycones

Due to the characteristic parent structure of 2-phenyl chromone, most of the fragment ions for the flavone skeleton were observed in the ESI-MS/MS spectra, and further fragment ions could be more informative for structural elucidation. In this way, Peaks **9**, **16**, **18**, **19**, **21**, **22**, **23**, **24**, **25**, **26** and **29** were tentatively identified as aglycones because of their similar fragmentation features of the ESI-MS/MS spectra, as shown in Table 1. In addition, several flavone derivatives of Peaks **27**, **28** and **32** were also observed.

Peak **9** presented the [M − H]^−^ ion at *m*/*z* 303 and further suffered neutral losses of 18 Da (H_2_O) and 28 Da (CO) of the C ring to produce the fragment ions of the [M − H − H_2_O]^−^ ion at *m*/*z* 285 and the [M − H − CO]^−^ ion at *m*/*z* 275. Typically, the ions at *m*/*z* 151 and *m*/*z* 125 correspond to the characteristic retro-Diels–Alder (RDA) cleavage between different chemical bonds of the C ring. In addition, fragment ions of [M − H − H_2_O − 44]^−^ at *m*/*z* 241, [M − H − CO − 58]^−^ at *m*/*z* 217 and [M − H − CO − 58 − 18]^−^ at *m*/*z* 199 were probably produced by the successive neutral loss of the CO moiety, the CO + CH_2_O moiety and the H_2_O moiety. Besides, the fragment ion at *m*/*z* 175 was regarded as the neutral loss of the B ring moiety from [M − H − H_2_O]^−^ at *m*/*z* 285. Based on the comparisons of the proposed fragmentation pathways and the MS/MS spectra in the previous study [27], Peak **9** was identified as taxifolin (calculated for C_15_H_12_O_7_, *m*/*z* 304). Similarly, Peak **16** (ESI-MS: [M − H]^−^ at *m*/*z* 287) shared the same basic parent skeleton of dihydroflavonol with Peak **9** and was identified as aromadendrin (calculated for C_15_H_12_O_6_, *m*/*z* 288) due to the absence of hydroxyl group (-OH) at the C-3′ position of the B ring [12].

Peak **24** exhibited the deprotonated molecular ion at *m*/*z* 271, with MS/MS fragment ions at *m*/*z* 185, 151, 125 and 119. Due to the characteristic fragment ions at *m*/*z* 151, 125 and 119 produced by the RDA cleavage of the C ring, Peak **24** was thus identified as naringenin (calculated for C_15_H_12_O_5_, *m*/*z* 272) [7]. Sharing the same basic parent skeleton of dihydroflavone with Peak **24**, Peak **29** was identified as sakuranetin (calculated for C_16_H_14_O_5_, *m*/*z* 286), which was indicated by a deprotonated molecular ion [M − H]^−^ at *m*/*z* 285 in the negative mode, with several fragment ions, such as [M − H − CH_3_]^−^ at *m*/*z* 270, [M − H − CO − C]^−^ at *m*/*z* 243 and [M − H − C_8_H_8_O]^−^ at *m*/*z* 165. Additionally, the ion at *m*/*z* 119 corresponded to the characteristic RDA-cleavage of the C ring and further to the dissociation of the acetenyl moiety (*m*/*z* 93). Based on the comparison of the proposed fragmentation pathways and the MS/MS spectra of the previous study, Peak **29** was suggested as sakuranetin [28].

Peak **27** showed the [M − H]^−^ ion at *m*/*z* 325 and its fragment ions at *m*/*z* 271, 185 and 125, which coincided with Peak **24** and presumably was identified as a naringenin derivative. For Peak **28** ([M − H]^−^ at *m*/*z* 551), those MS/MS fragment ions at *m*/*z* 285, 165 and 119 were identical to those of Peak **29**. Tentatively, the precursor ion at *m*/*z* 551 was formed by two sakuranetin with the further neutral losses of H_2_O and H_2_. Hence, Peak **28** was determined to be the sakuranetin dimer, which was found in *R. davurica* for the first time in this study. However, previous studies revealed similar carbon-carbon interflavanoid linkage types, such as 3,8’-coupled dimer of sakuranetin [29], chamaejasmin and 3”-epidiphysin [30].

For flavones, Peaks **18** ([M − H]^−^ at *m*/*z* 285), **19** ([M − H]^−^ at *m*/*z* 301), **21** ([M − H]^−^ at *m*/*z* 269) and **22** ([M − H]^−^ at *m*/*z* 285) were identified as luteolin (calculated for C_15_H_10_O_6_, *m*/*z* 286), quercetin (calculated for C_15_H_10_O_7_, *m*/*z* 302), apigenin (calculated for C_15_H_10_O_5_, *m*/*z* 270) and kaempferol (calculated for C_15_H_10_O_6_, *m*/*z* 286), respectively. They were further confirmed by comparing the retention time and the MS/MS spectra with the corresponding standards. Peaks **23** and **25** had the same deprotonated molecular ion at *m*/*z* 301 and some other characteristic fragment ions, such as the ions at *m*/*z* 151 and *m*/*z* 125 produced by the RDA cleavage of C ring, the neutral loss of H_2_O at *m*/*z* 283 and the dissociation of B ring at *m*/*z* 179. Hence, Peaks **23** and **25** were tentatively identified as the isomers of Peak **19**. As for Peak **26**, the [M − H]^−^ ion at *m*/*z* 299 was obtained, which further generated MS/MS fragment ions [M − H − CH_3_]^−^ at *m*/*z* 284, [M − H − CH_3_ − CO]^−^ at *m*/*z* 256, [M − H − CH_3_ − CO_2_]^−^ at *m*/*z* 240 and [M − H − CH_3_ − CO − CHO]^−^ at *m*/*z* 227. Compared with those ESI-MS/MS spectra and fragmentation pathways, Peak **26** was thus identified as rhamnocitrin (calculated for C_16_H_12_O_6_, *m*/*z* 300) [11,31]. Peak **32** presented the [M − H]^−^ ion at *m*/*z* 359, and its MS/MS fragment ions at *m*/*z* 285, 267, 241 and 223 were similar to that of Peak **18**, which suggested Peak **32** as a luteolin derivative.

#### 2.2.2. Identification of Flavonoid Glycosides

According to Table 1, flavonoid conjugates account for a large proportion of the components detected in the extract of *R. davurica*. For a better illustration, we classified them into two groups: *C*-glycosides and *O*-glycosides, which were identified by comparing their MS/MS spectra with those of corresponding standards and the literature reported previously.

**Figure 3:**
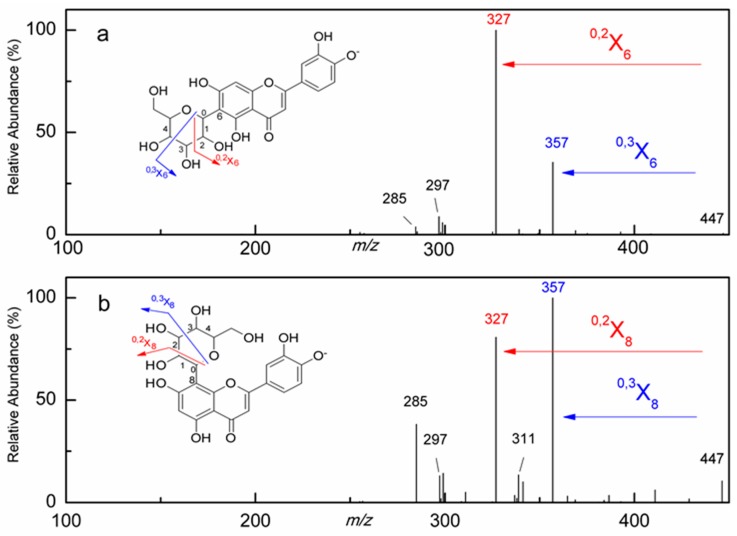

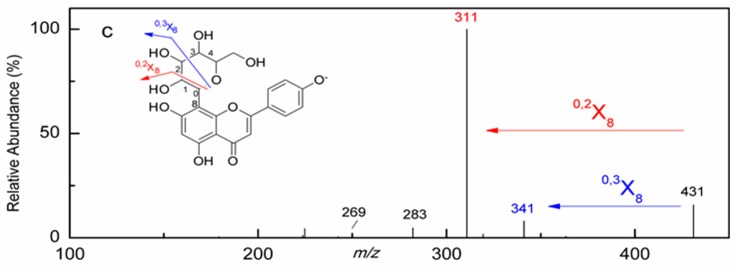
The MS/MS spectra and proposed fragmentation pathways of representative *C*-glycosides of isoorientin (**a**) (Peak **2**), orientin (**b**) (Peak **1**) and vitexin (**c**) (Peak **3**). The peak numbers in this figure correspond to those used in Table 1.

As for *C*-glycosides from *R. davurica*, the MS/MS spectra and proposed fragmentation pathways are shown in Figure 3. Specifically, fragments [M − H − 60]^−^, [M − H − 90]^−^ and [M − H − 120]^−^ are the characteristic diagnostic ions of glycone [1]. Peaks **1** and **2** showed the same [M − H]^−^ ion at *m*/*z* 447 and MS/MS spectra at *m*/*z* 357, 327, 297 and 285, implying that they were structural isomers. Fragment ions at *m*/*z* 357 and 327 were due to the collision-induced dissociation (CID) of glycone at the 0−3 bond (^0,3^X, 90 Da) and the 0−2 bond (^0,2^X, 120 Da). Taken into consideration both the deprotonated molecular ions at *m*/*z* 447 and the aglycone ion at *m*/*z* 285 ([M − H − 162]^−^, loss of a hexose moiety), they were tentatively characterized as luteolin monohexoside. For Peak **1** (Figure 3b), the diagnostic ion of base peak for *C*-8-hexosides of [M − H − 90]^−^ at *m*/*z* 357 (^0,3^X_8_) was more abundant than that of [M − H − 120]^−^ at *m*/*z* 327 (^0,2^X_8_). However, for Peak **2** (Figure 3a), the base peak of [M − H − 120]^−^ at *m*/*z* 327, characteristic of *C*-6-hexosides (^0,2^X_6_), was of higher abundance than that of [M − H − 90]^−^ at *m*/*z* 357 (^0,3^X_6_). Hence, Peaks **1** and **2** were identified as orientin (calculated for C_21_H_20_O_11_, *m*/*z* 448, luteolin 8-*C*-glucopyranoside) and isoorientin (luteolin 6-*C*-glucopyranoside) [1], both of which were found in *R. davurica* for the first time in this study. As expected, our results were in accordance with the previous studies showing that *C*-8-glycoside eluted before its *C*-6-isomer [32,33]. Similarly, Peak **3** (Figure 3c) exhibited the [M − H]^−^ ion at *m*/*z* 431 and the aglycone ion at *m*/*z* 283 ([M − H − 162]^−^, loss of a hexose moiety). With the higher abundant peak of [M − H − 90]^−^ at *m*/*z* 341, characteristic of *C*-8-hexosides (^0,2^X_8_), and further MS/MS ions at *m*/*z* 283 and 269, Peak **3** was thus identified as vitexin (calculated for C_21_H_20_O_10_, 432 Da, apigenin 8-*C*-glucopyranoside) [3]. Finally, Peaks **1**, **2** and **3** were further validated by comparing their LC-MS/MS with the corresponding standards.

The fragmentation pathways of *O*-glycosyl flavonoids in the negative ion mode (NI) are characterized not only by the loss of the sugar moieties, but also by the deprotonated aglycone species ([Y_0_]^−^) and the radical aglycone ions ([Y_0_ − H]^−^) [4]. At the same time, most flavonoid glucosides commonly showed MS^1^ spectra; this phenomenon was due to the rearrangement reaction of the charge-remote neutral loss of a glycosyl residue and followed by the protonation at the C-3 carbonyl group [23].

Peaks **4** and **11** exhibited the same [M − H]^−^ ion at *m*/*z* 463 and the aglycone ion at *m*/*z* 301 ([M − H − 162]^−^, loss of a hexose moiety), indicating that they were quercetin monosaccharides. Additionally, the aglycone ions of quercetin showed distinct relative abundances of [Y_0_]^−^ at *m*/*z* 301 and [Y_0_ − H]^−^ at 300. By comparing its LC-MS/MS with the standard compound of isoquercitrin, Peak **4** was identified as isoquercitrin (calculated for C_21_H_20_O_12_, 464 Da, quercetin 3-*O*-glucoside). Peak **11** could thus be deduced as quercetin 7-*O*-glucoside according to a previous study [2] and firstly identified in *R. davurica* in this work. Peaks **6** and **17** showed the same deprotonated molecular ion at *m*/*z* 447. Besides, the aglycone fragment ions at *m*/*z* 285 indicated the loss of a hexose moiety ([M − H − 162]^−^). Considering the MS/MS fragments of [Y_0_]^−^ at *m*/*z* 285 and [Y_0_ − H]^−^ at 284, Peaks **6** and **17** were identified as astragaloside (calculated for C_21_H_20_O_11_, 448 Da, kaempferol 3-*O*-glucoside) and kaempferol 7-*O*-glucoside [2], and both of them were found in *R. davurica* for the first time in this study. Furthermore, Peak **6** was validated by the corresponding standard. Peak **5** gave the deprotonated molecular ion at *m*/*z* 461 with a [Y_0_]^−^ at *m*/*z* 299 (the loss of a hexose moiety), suggesting that it was a diosmetin monosaccharide. Meanwhile, the presence of the ion at *m*/*z* 284 was yielded due to the loss of the methyl moiety, and its glycosylation took place at C-7. Therefore, Peak **5** was tentatively identified as diosmetin 7-*O*-glucoside [4]. Peak **7** also showed the [M − H]^−^ ion at *m*/*z* 447 and the aglycone ion at *m*/*z* 285 ([M − H − 162]^−^, the loss of a hexose moiety); while the neutral losses of 120 Da and 146 Da of MS/MS fragments at *m*/*z* 327 and 301 indicated that Peak **7** was luteolin 5-*O*-glucoside [3]. The two glucosides were also identified in *R. davurica* for the first time in this work.

#### 2.2.3. Identification of Anthraquinones and Their *O*-glycosides

Peak **10** exhibited the deprotonated molecular ion at *m*/*z* 445 with a [Y_0_]^−^ at *m*/*z* 283 (loss of a hexose moiety). The ion at *m*/*z* 268 corresponds to the loss of a methyl moiety (15 Da). By comparing its MS/MS spectra with a reported study, Peak **10** was tentatively identified as physcion 8-*O*-glucoside [34]. For Peak **31** ([M − H]^−^ at *m*/*z* 283), several fragment ions at *m*/*z* 268, 239, 211 and 195 were obtained. Fragment ions of [M − H − 15]^−^ at *m*/*z* 268, [M − H − 15 − 29]^−^ at *m*/*z* 239 and [M − H − 15 − 29 − 44]^−^ at *m*/*z* 195 were produced by the successive loss of the methyl moiety (15 Da), neutral CO + H moiety (29 Da) and CO_2_ moiety (44 Da). Besides, the fragment ion of at *m*/*z* 211 was regarded as the neutral loss of CO moiety (28 Da) from [M − H − CH_3_ − COH]^−^ at *m*/*z* 239. As a result, Peak **31** was determined to be physcion (calculated for C_16_H_12_O_5_, 284 Da), whose fragment ions were also consistent with those observed using ESI-MS/MS [35]. With the same [M − H]^−^ ion at *m*/*z* 283 in the negative ESI-MS/MS spectrum (Table 1), Peak **30** was identified as question in accordance with a C_16_H_12_O_5_ formula, considering the retention times, LC profile with C18 column and the similar CID fragmentation pathways reported for the two isomers [36].

#### 2.2.4. Others

Peak **12** showed a precursor ion at *m*/*z* 195 and fragment ions at m/z 167, 152, 136 and 108, which were similar to the MS/MS data of Iodolactone, and was thus tentatively deduced as an iodolactone derivative [37]. As for Peak **20**, the [M − H]^−^ ion at *m*/*z* 169 gave a series of product ions at *m*/*z* 151, 125, 107, 83 and 57 due to the consecutive neutral loss of H_2_O (18 Da), C_2_H_2_ (26 Da), CO (28 Da), C_2_ (24 Da) and C_2_H_2_ (26 Da). Hence, Peak **20** was identified as oxireno[4,5]cyclopenta[1,2-*c*]pyran, which was similar to catalpol aglycone [38]. Moreover, the two compounds above were firstly found in *R. davurica*.

### 2.3. Quantification of Flavonoids in R. davurica

Table 2 shows the results of method validation for the three representative standards, of which the calibration curves exhibited good linearity at 360 nm (R^2^ ≥ 0.9990). In total, 32 components can be classified into five groups based on the structural identifications above. Hence, the individual component contents in *R. davurica* were quantified by using the corresponding standards for calibration for each group (kaempferol for flavone aglycones, vitexin for flavonoid *C*-glycosides, rutin as an external reference sample for flavonoid *O*-glycosides, others and unknown), and their corresponding concentrations were calculated as shown in Table 1.

**Figure 4:**
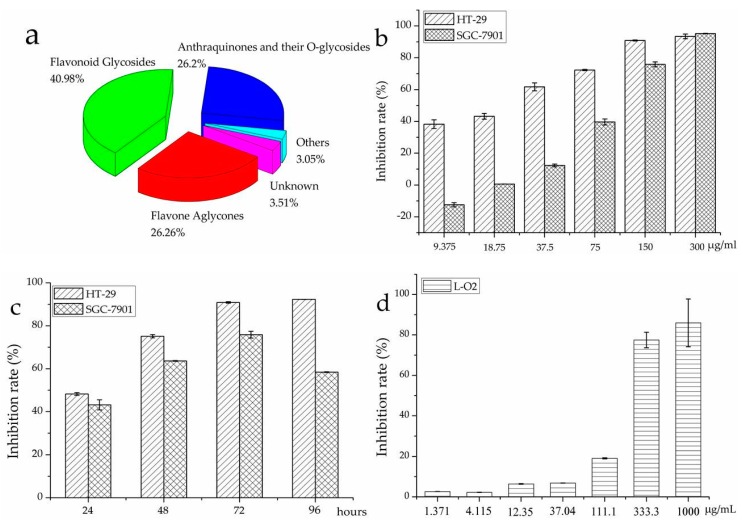
The percentage compositions of different component groups (**a**) from *R. davurica* and its dose-dependent (**b**) and time-dependent (**c**) antiproliferative activities against HT-29 and SGC-7901 cells and its general toxicity on L-O2 cells (**d**).

According to the HPLC screening data summarized in Figure 4a, flavonoids accounted for the majority of the chemical compositions and represented the dominant class of components (67.24%) in *R. davurica*. Due to the chemical diversities of different groups, flavonoid glycosides exhibited higher content of 40.98%, followed by the flavone aglycones of 26.26%, anthraquinones and their *O*-glycosides of 26.2%, respectively. In general, vitexin was the most abundant glycoside with 86.07 mg/100 g DW (dry weight); aromadendrin and question, the major components of aglycones and anthraquinones, accounted for 21.48 mg/100 g DW and 42.17 mg/100 g DW. In addition, the other components in trace quantities also showed significant variance in the constitutes shown in Table 1. Therefore, the discrepancies in the diversities of the chemical compositions and their corresponding contents could further affect the quality control and future medical exploration of *R. davurica*.

### 2.4. Antiproliferation Assays on R. davurica

The bioactive evaluations in one plant species depend on both the qualitative and quantitative knowledge on this species [39]. As we know, *R. davurica* has long been used as a kind of folk remedy in many Asian countries. According to the quantification above, flavonoids accounted for the majority of the chemical compositions and represented the dominant class of components in *R. davurica*. Flavonoids ubiquitously exist and are widely consumed as the important secondary metabolites from natural plants and have remarkable anticancer activities. However, there is very little study on their anticancer activities.

In this study, for the first time, we focused on the antiproliferative effects of *R. davurica* on human cancer cell lines, like HT-29 and SGC-7901 cells. As shown in Figure 4b, it is found out that *R. davurica* exhibited significant dose-dependent antiproliferative activities against HT-29 and SGC-7901 cells with IC_50_ values of 24.96 ± 0.74 and 89.53 ± 4.11 μg/mL, respectively. Meanwhile, Figure 4c shows that the inhibitory activities against both HT-29 and SGC-7901 cells significantly increased by the treatment with *R. davurica* in a time-dependent manner from 24 h–96 h at a dose of 150 μg/mL, although there was a decrease on SGC-7901 cells at the time from 72 h–96 h. Considering the drug safety, the evaluation of the general toxicity of *R. davurica* on the normal human hepatic cells (L-O2) was also conducted (Figure 4d). As expected, *R. davurica* displayed a much higher IC_50_ at 229.19 ± 8.52 μg/mL on L-O2, which suggested that *R. davurica* showed very low or no toxicity on hepatic cell viability.

To some extent, the efficacy of the folk medicine is deemed to rely heavily on the characteristics of its complex chemical components, which could further lead to the complicated and diverse pharmacological mechanisms. Accordingly, it is important to figure out the correlations between the antiproliferative activities and the contents of the corresponding chemical compositions. Our phytochemical investigations on *R. davurica* have revealed that flavonoids accounted for the prominent constituents in diversity and content and could be the most promising antiproliferative components of *R. davurica*.

## 3. Materials and Methods

### 3.1. Chemicals and Reagents

The reference standards of rutin, orientin, isoorientin, vitexin, isoquercitrin, astragaloside, luteolin, apigenin, quercetin and kaempferol were purchased from Shanghai Tauto Biotech (Shanghai, China). Formic acid (FA) and acetonitrile (ACN) of HPLC grade were provided by TEDIA Company Inc. (Fairfield, OH, USA). Fetal bovine serum (FBS) and Dulbecco’s Modified Eagle Medium (DMEM) were obtained from Gibco (Life Technologies, Grand Island, NY, USA). Sulforhodamine B (SRB), glutamine, trichloroacetic acid, streptomycin, penicillin and DMSO were purchased from Sigma-Aldrich (St. Louis, MO, USA). Polyamide was obtained from an industrial chemical company affiliated to Nan Kai University (Tianjin, China). Water for HPLC-UV and ESI-MS/MS was prepared with EPED (Nanjing Yeap Esselte Technology Development Co., Nanjing, China). All other chemicals and solvents were of analytical grade.

### 3.2. Plant Materials

The barks of *R. davurica* originated from the northeast of China and were provided by Jikang Pharmaceutical Co., Ltd. (Baoding, China). Those raw materials were dried at 40 °C and powdered using a high speed disintegrator, then packed in sealed polyethylene bags and stored in a refrigerator at 4 °C until use. The authentication and identification of the specimens was kindly assisted by the taxonomist (Guangwan Hu) of Key Laboratory of Plant Germplasm Enhancement and Specialty Agriculture (Wuhan Botanical Garden), Chinese Academy of Sciences. A voucher specimen (No. 0031) was deposited in the herbarium of the Key Laboratory.

### 3.3. Sample Preparation

First, 100 g raw powered sample of *R. davurica* were accurately weighed and then extracted in an ultrasonic bath (KQ-3200DE, 300 × 150 × 150 mm, SHUMEI, Kunshan, China) with 60% ethanol for 30 min at room temperature, and the residue was re-extracted twice as described above. Next, the extracts were combined and filtered. After that, the filtrates were concentrated in a rotary evaporator under reduced pressure at 40 °C to afford the crude syrup residues. Later, the crude extracts were dispersed in water (100 mL) and subjected to liquid-liquid partition with petroleum ether (PE, b.p. 60–90 °C, to remove chlorophyll) and ethyl acetate (EA), successively. Afterwards, the EA layers were concentrated and fractionated by a polyamide column (45 cm × 5.3 cm). The column was eluted with distilled water to nearly colorless to remove water soluble impurities [26] and then with 80% ethanol to give the expected fractions. Finally, the fractions were concentrated and lyophilized in a freeze dryer to dryness, and the residues were stored at 4 °C for the subsequent analysis.

### 3.4. Antiproliferation Assays in Vitro

#### 3.4.1. Cell Culture

Human cancer cell lines of HT-29 (colon carcinoma) and SGC-7901 (gastric carcinoma) were obtained from the China Center for Type Culture Collection (CCTCC). Cells were routinely grown in DMEM medium supplemented with 10% fetal bovine serum (FBS), glutamine (2 mM) and 1% penicillin (100 U/mL)-streptomycin (100 µg/mL). Furthermore, the cell lines were sub-cultured twice a week, and incubated in a humidified atmosphere with 5% CO_2_ and 90% relative humidity (RH) under 37 °C. The number of living cells was assessed using a hematocytometer and phase-contrast microscopy. Finally, the cells over 80% confluence (growth phase) were used for the following cell antiproliferation assay [40].

#### 3.4.2. Sulforhodamine B Antiproliferation Assays

The antiproliferative activities against HT-29 and SGC-7901 cells were estimated by means of the protein-staining sulforhodamine B (SRB) microculture colorimetric assay with some modification [41]. The assay shows high sensitivity to total cellular protein content and linearity to cell density, which has been used for in vitro anticancer evaluation at the National Cancer Institute (Bethesda, MD, USA) [42]. Briefly, a 100-μL cell suspension of the trypsinized monolayer cells in DMEM medium was seeded into 96-well plates with a density of 1.0 × 10^4^ cells per well. After incubation at 37 °C in 5% CO_2_ and 90% relative humidity for 24 h to resume exponential growth and stabilization, the culture medium was removed carefully, and an aliquot of 100 μL *R. davurica* extracts was added into each well in the plates. All of the test samples were first dissolved in DMSO and further diluted with the medium to the final DMSO content less than 0.1%, which was innocuous on cell growth and proliferation. After incubation for another 72 h, cells were fixed with 50 μL 10% cold (4 °C) trichloroacetic acid (TCA) for 30 min at 4 °C. Afterwards, the supernatants were washed out with deionized water five times and air dried at room temperature. Subsequently, the dried plates were stained with 100 μL 4 mg/mL SRB in 1% acetic acid solution for 30 min at room temperature. Next, the plates were washed five times with 1% acetic acid to remove the unbound SRB and then air dried overnight. The protein-bound SRB was solubilized with 150 μL of 10 mM Tris base (pH 10.5), and the plates were left on a gyratory shaker for 10 min. The complete medium with less than 0.1% DMSO was used as the control. The optical density (OD) value of each well was determined with a 96-well plate reader (Tecan) at a wavelength of 540 nm. The inhibition rate (%) was calculated using the equation: inhibition = (ODC − ODT)/ODC × 100%, where ODC and ODT were the OD values of controls and samples of *R. davurica*, respectively. Each test sample solution was performed in triplicate, and the results were expressed as the means ± SD (standard deviation).

### 3.5. HPLC-ESI-MS*/*MS Analysis of R. davurica

#### 3.5.1. HPLC Fingerprint Analysis

Experiments for phytochemical fingerprint analysis of *R. davurica* were carried out using a Thermo Accela 600 HPLC system (Thermo Fisher Scientific, Waltham, MA, USA). The chromatographic separation was performed on a Waters SunFire™ RP-C18 column (150 mm × 4.6 mm, 3.5 μm). The mobile phases were composed of 0.1% aqueous formic acid (A) and acetonitrile (B). An aliquot of a 10-μL sample solution was injected into the HPLC system, and the linear eluting gradient was as follows: 20% B in 0–2 min, 20%–45% B in 2–15 min, 45%–70% B in 15–31 min and 70% B in 31–35 min. The column temperature was maintained at 30 °C. The flow rate was 0.4 mL/min, and the online UV spectrum was monitored at the wavelength of 360 nm.

#### 3.5.2. ESI-MS/MS Analysis

The ESI-MS/MS analysis was carried out using a TSQ Quantum Access MAX mass spectrometer (Thermo Fisher Scientific) equipped with an ESI source operating in Auto-MS^n^ mode to obtain fragmentation. The negative ionization mode was applied, and the optimized instrument settings were set as follows: source voltage, 3.0 kV; cone voltage, 40.0 V; desolvation temperature, 350 °C; capillary temperature, 250 °C; nebulizing gas flow rate, 6.0 L/min; sheath gas (N_2_) pressure, 40 arb; Aux gas (N_2_) pressure, 10 arb; collision energy (CE), 10 V; collision energy grad (CE grad), 0.035 V/m. Mass spectra data were obtained with the full-scan mode for *m*/*z* in the range from 150 to 1500, and the nine most abundant ions were selected for the further MS^2^ spectra. All data acquisition and analysis were performed using the Thermo Xcalibur ChemStation (Thermo Fisher Scientific).

### 3.6. Quantitative Analysis of Flavonoids Compounds

For the quantitative analysis, three representative standards (rutin, vitexin and kaempferol) were applied to calculate the individual component concentration present in *R. davurica* by HPLC. Each standard was accurately weighed, prepared in methanol, and then, the calibration curves were established by diluting the standard stock solutions into a series of concentrations measured at 360 nm. Six different concentrations (1.0–333 μg/L) of each standard were used for the calibrations and measured in triplicate. Method validations included accuracy, correlation determination (R^2^), limit of detection (LOD), limit of quantitation (LOQ), linearity range, repeatability, precision and recovery. Finally, the quantification of each individual compound was calculated based on the calibration curves of the standards for the corresponding HPLC peak area values of *R. davurica*. In the present study, individual components are presented as mg per 100 g DW, and the total content was defined as the sum of each corresponding quantified component.

## 4. Conclusions

In this study, a HPLC-ESI-MS/MS method was employed to comprehensively analyze the phytochemical fingerprint profile of *R. davurica*. As a result, 32 peaks were detected. Structural identifications and characterizations showed that 23 of them were identified as flavonoids based on the comparisons of their ESI-MS/MS data with the corresponding standards and fragmentation pathways reported in the previous literature. Among 32 components identified, including orientin (Peak **1**), isoorientin (Peak **2**), diosmetin 7-*O*-glucoside (Peak **5**), astragaloside (Peak **6**) and luteolin 5-*O*-glucoside (Peak **7**), 14 components were identified in *R. davurica* for the first time. For individual component quantitative analysis, flavonoid glycosides, flavone aglycones and anthraquinones exhibited higher content of 40.98%, 26.26% and 26.2%, respectively, which consisted of the major components of *R. davurica*. For the discrepant contents of individual components, vitexin, aromadendrin and question accounted for 86.07, 24.18 and 42.17 mg/100 g DW, respectively. Meanwhile, to better correlate the phytochemical components with their pharmacological activities, the antiproliferative activities against human cancer cells were tested in vitro and exhibited distinct inhibitory effects against HT-29 and SGC-7901 cells with IC_50_ values at 24.96 ± 0.74 and 89.53 ± 4.11 μg/mL, respectively. Finally, *R. davurica* displayed a much higher IC_50_ at 229.19 ± 8.52 μg/mL on L-O2, which suggested that *R. davurica* showed very low or no toxicity on hepatic cell viability. The current study revealed for the first time the correlations between the flavonoids of *R. davurica* with their antiproliferative activities, which indicated that the fingerprint profile of flavonoids and their anticancer activities could provide valuable information on the quality control for this herbal medicine and its derived natural remedies.

## Figures and Tables

**Table 1:** Identification and quantitative analysis of flavonoids and other compounds corresponding to the chromatographic peaks in Figure 1 by HPLC-ESI-MS/MS.

Peak	Rt (min)	[M − H]^−^ (*m*/*z*)	MS^2^ (*m*/*z*)	Identifications	Contents (mg/100 g)
**1**	7.72	447	447, 357, 327, 311, 297, 285	Orientin ^a^	1.43
**2**	9.26	447	447, 357, 327, 297, 285	Isoorientin ^a^	2.87
**3**	11.20	431	431, 341, 311, 283, 269	Vitexin ^a^	86.07
**4**	12.10	463	463, 301, 300	Isoquercitrin ^a^	2.76
**5**	12.70	461	461, 299, 284	Diosmetin 7-*O*-glucoside ^b^	2.66
**6**	13.27	447	447, 285, 284	Astragaloside ^a^	2.19
**7**	14.06	447	447, 327, 301, 300, 285, 270	Luteolin 5-*O*-glucoside ^b^	4.07
**8**	14.32	1017	1016, 903, 790, 677, 564, 451, 338, 225	Unknown	2.50
**9**	14.63	303	303, 285, 275, 241, 217, 199, 175, 151, 125	Taxifolin ^b^	15.51
**10**	15.24	445	445, 283, 268	Physcion 8-*O*-glucoside ^b^	3.40
**11**	15.68	463	463, 301, 300	Quercetin 7-*O*-glucoside ^b^	3.08
**12**	16.14	195	195, 167, 152, 136, 108	Iodolactone derivative ^b^	4.00
**13**	16.57	665	665, 470, 357, 338, 243	Unknown	2.49
**14**	17.27	553	553, 469, 425, 355, 243	Unknown	1.62
**15**	17.49	509	509, 449, 421, 359, 341, 315, 271, 239	Unknown	2.70
**16**	18.55	287	287, 269, 259, 243, 215, 201, 151, 125	Aromadendrin ^b^	21.48
**17**	19.04	447	447, 285, 183, 165, 119, 93	Kaempferol 7-*O*-glucoside ^b^	3.70
**18**	19.49	285	285, 267, 241, 217, 199, 175, 151, 133	Luteolin ^a^	2.97
**19**	19.87	301	301, 273, 229, 179, 151, 121, 107	Quercetin ^a^	1.17
**20**	21.33	169	169, 151, 125, 107, 83, 57	Oxireno[4,5]cyclopenta[1,2-*c*]pyran ^b^	4.10
**21**	22.28	269	269, 241, 225, 201, 181, 159, 133	Apigenin ^a^	3.47
**22**	22.64	285	285, 257, 241, 229, 213, 185, 151, 107, 93	Kaempferol ^a^	11.77
**23**	23.02	301	301, 283, 245, 227, 151, 125	Quercetin isomer	7.29
**24**	23.99	271	271, 185, 151, 125, 119	Naringenin ^b^	0.89
**25**	24.81	301	301, 283, 179, 165, 135, 109	Quercetin isomer	0.08
**26**	25.89	299	299, 284, 283, 256, 255, 240, 227	Rhamnocitrin ^b^	1.62
**27**	26.90	325	325, 307, 289, 271, 263, 185, 169, 137, 125	Naringenin derivative	0.05
**28**	28.09	551	285, 179, 165, 119	Sakuranetin dimer	0.22
**29**	29.44	285	285, 270, 243, 165, 151, 119, 93	Sakuranetin ^b^	2.95
**30**	29.88	283	285, 270, 243, 165, 151, 119, 93	Questin ^b^	42.17
**31**	30.49	283	283, 268, 267, 239, 211	Physcion ^b^	24.00
**32**	32.15	359	359, 285, 267, 241, 223	Luteolin derivative	0.27

^a^ Identified with the corresponding standards; ^b^ identified based on the reported literature. Each individual component is expressed as mg per 100 g DW. Rt, retention time.

**Table 2:** Liner equation, correlation coefficients, limits of detection and limits of quantification of three standards.

Compound	Liner Equation	R^2^	LOD (μg/mL)	LOQ (μg/mL)	Linear Range (μg/mL)
Rutin	*Y* = 31.794*x* − 24.028	0.9992	0.30	1.0	1.0–333
Vitexin	*Y* = 33.007*x* + 42.294	0.9991	0.30	1.0	1.0–333
Kaempferol	*Y* = 78.286*x* + 79.191	0.9990	0.1	0.33	0.33–333

LOD = limit of detection, S/N = 3; LOQ = limit of quantitation, S/N = 10.
